# Modification of the EPA method 1631E for the quantification of total mercury in natural waters

**DOI:** 10.1016/j.mex.2020.100987

**Published:** 2020-07-08

**Authors:** Antonella Tassone, Sacha Moretti, Maria Martino, Nicola Pirrone, Francesca Sprovieri, Attilio Naccarato

**Affiliations:** CNR-Institute of Atmospheric Pollution Research, Division of Rende, UNICAL-Polifunzionale, I-87036 Arcavacata di Rende, CS, Italy

**Keywords:** Mercury, Environmental waters, Contamination issues, Reagent blank, CV-AFS, Sample preparation

## Abstract

To support the effectiveness of the Minamata Convention, the accurate determinations of mercury (Hg) in natural waters is an important but certainly challenging task due to the low concentrations expected in ambient samples. Mercury contamination may occur from many sources such as the unproperly-cleaning of storage bottles or the use of reagents for sample analysis with Hg traces, thus leading the analyst to easily run into errors. In our work, we propose some key modifications to the United States Environmental Protection Agency(EPA) method 1631E aimed at reducing the Hg contamination of reagents, storage containers, and minimizing the carryover effect in the instrumental line of sampling. The changes introduced have been tailored for the use of the method with cold vapor atomic fluorescence spectroscopy (CV-AFS) instrumentation and tested as part of a United Nations Environment Program (UNEP) ring test. Although the edited method was tested with natural water samples, the proposed method improvements can also apply to the Hg analysis in solid matrices that require the prior acid digestion of the samples.

• A customized version of the EPA method 1631E is proposed for the analysis of aqueous samples.
• New protocols for the reduction of contamination in the storage bottles and reagents used for the preparation of BrCl solution are provided.
• A useful strategy for the control of the memory effect is included.

## Graphical Abstract

**Figure:**
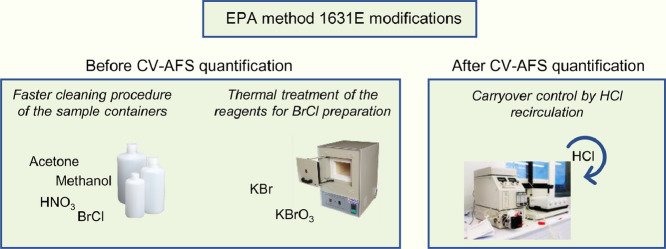

**Specifications Table**

**Table:** 

Subject Area:	Chemistry
More specific subject area:	Analytical Chemistry
Method name:	US - EPA method 1631E
Name and reference of original method:	U.S. Environmental Protection Agency Method 1631 “Mercury in water by oxidation, purge and trap, and cold vapor atomic fluorescence spectrometry” revision E
Resource availability:	https://www.epa.gov/sites/production/files/2015-08/documents/method_1631e_2002.pdf

## Method details

### Overview

Mercury (Hg) is a toxic contaminant, which due to its high mobility and long residence time in the environment poses a threat of global concern for both human health and ecosystems [1]. Indeed, Hg is continuously cycled and re-cycled as a consequence of natural and anthropogenic activities, which are responsible for its occurrence in the atmosphere, but also aqueous compartments and biota. Its reliable monitoring has been committing a part of the scientific community focused on the characterization of the ambient matrices and control of the pollutants which are potentially harmful to humans and the environment [2], [3], [4]. A comprehensive assessment of Hg risk involves not only a variety of analytical methods, which depend on the chemical species of mercury to be assayed and the type of matrix, but also involves the support of modeling studies, useful for an in-depth understanding of Hg fate and transport [5,6].

In this context, of particular importance is the Hg quantification in the aquatic environments, which are too often affected by Hg contamination despite their key role in ecological webs and the strong potential impact on human health. Determination of mercury in natural waters requires strict precautions since low levels, down to ng L^−1^, can be expected, especially in unpolluted areas. In practice, every stage of analysis, from the sampling to the quantification, requires scrutiny for the attainment of reliable results. In this regards, specific instructions and recommendations for each of these stages are included in the method “Mercury in water by oxidation, purge-and-trap, and cold vapor atomic fluorescence spectrometry” (EPA 1631E), issued by the United States Environmental Protection Agency (US-EPA) and worldwide used for the quantification of total mercury (THg) in natural waters, e.g., seawater, freshwater, rainwater [7]. This analytical method relies on three different steps: (i) chemical oxidation of Hg, (ii) purge-and-trap of gaseous Hg after chemical reduction, and (iii) detection by cold vapor atomic fluorescence spectroscopy (CV-AFS) for THg quantification. More detailed, aqueous samples are first oxidized using bromine monochloride (BrCl) to convert the mercury present as different species into its inorganic water-soluble form (Hg^2+^). Once the samples are completely oxidized, as suggested by a permanent yellow color of the solution, 0.2% v/v hydroxylamine hydrochloride (NH_2_OH·HCl) is added as a mild reducing agent to destroy free halogens. Then, the samples are further reduced with 3% v/v stannous chloride (SnCl_2_·2H_2_O), to obtain mercury in its elemental and volatile state (Hg^0^), which can be easily removed from the solution by purging the sample or using a phase separator (PS). The gaseous Hg^0^ is collected on a gold trap system, which simultaneously allows for the removal of interfering volatile compounds and the Hg preconcentration. Subsequently, the loaded Hg is desorbed at 550°C and the generated Hg^0^, is transferred to an optical cell for detection via atomic fluorescence. The use of CV-AFS as determination technique makes the whole method highly sensitive and selective, thus resulting in a low detection limit (down to ng L^−1^ level), wide linear calibration range as well as high accuracy and precision at low concentration values [8]. On the other hand, a potential drawback of such a sensitive approach is the responsiveness to contamination, an especially important issue that must be addressed for reliable analysis at low Hg concentration levels, as those which involve water samples of background areas. This problem is commonly encountered because several sources can affect the result of the measurements, for example the improper cleaning of the storage bottles or the contamination of reagents used for the sample preparation and analysis. In this regard, each sample flask, as well as the working environment, must be thoroughly cleaned. Besides, a major issue could arise from the contamination during the sampling step, which would unavoidably affect the subsequent analyses and for this reason, strict precautions involving the material (usually FLPE) of the sample container must be taken.

This work proposes some customizations and technical improvements to the EPA method 1631E, focusing on the potential contamination issues during sampling and analysis of aqueous matrices.

### Chemicals and materials

The study was carried out using high-quality grade reagents, properly produced to avoid metal contamination of blanks and samples. Mercury (II) standard solutions were prepared by diluting a 1000 mg L^−1^ mercury stock solution (VWR Prolabo Chemical, France) at different levels in the range 0.5 – 100 ng L^−1^. For the BrCl preparation, we tested potassium bromate and potassium bromide released with four different purity trademarks:

• KBrO_3_ (EMSURE^Ⓡ^, Merck) and KBrO_3_ (ACS, Fluka).
• KBr (Suprapur^Ⓡ^, Merck) and KBr (ACS, Sigma Aldrich).

Both the reducing agents needed for the analysis, i.e., Tin(II) chloride dihydrate and hydroxyl-ammonium chloride, were put on the market from Merck with ACS quality, while the hydrochloric acid was of ultra-purity grade (Romil, UpA™). Ultrapure water (resistivity > 18.2 MΩ cm) used for the preparation of all aqueous solutions was obtained with a Millipore Purification Water System (Millipore Co., MA, USA). Pure argon (> 99.999%) used as a mercury-free purging carrier was further purified with a gold trap before the instrumental inlet.

For the cleaning of FLPE sampling bottles, we used acetone (Pestanal, Fluka), methanol (Pestinorm, VWR Prolabo Chemical) and an aqueous solution of nitric acid (3.5% v/v) obtained by dilution of nitric acid 67% (Normatom^Ⓡ^ for trace metal analysis, VWR Prolabo Chemical).

### Instrumentation

The analysis of the aqueous samples was carried out by a mercury analyzer Tekran 2600 (Tekran, Canada), which utilizes CV-AFS for the detection of the total mercury in liquid samples. This system is equipped with an autosampler (Tekran unit 2620) and a peristaltic pump module (Tekran unit 2610). Briefly, when an analysis is running, the liquid sample previously oxidized with the BrCl solution flows throughout the tubing system until it is mixed with the aqueous solution of SnCl_2_, which acts as a reducing agent and converts the oxidized mercury to the volatile elemental specie (Hg^0^). A key component of the instrument is the phase separator (PS) which allows for the removal of the gaseous mercury from the liquid phase using a countercurrent argon flow and its conveyance towards the trap system. This component, together with the tubing system, is a major source of memory effect because it is made of frosted glass on whose surface undesired deposits are easily formed as a result of the extended contact with the samples. For this reason, in operating procedures, it is recommended the frequent replacement of the tubing system and the thorough cleaning of the PS after analysis.

### Prevention from the contamination of sampling equipment

**Fig. 1:**
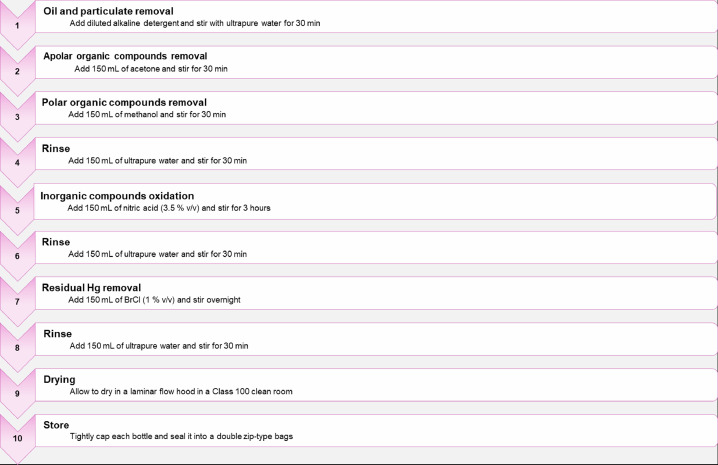
Customized protocol for the cleaning of FLPE sampling bottles.

A key point for an accurate determination of mercury in water at trace levels is the selection of the material of the sample container because it is fundamental to prevent the risk of contamination. Previous studies demonstrated that Fluorinated High-Density Polyethylene FLPE (e.g., Nalgene bottles) is suitable to avoid Hg contamination of samples during storage as well as losses due to Hg adhesion on the bottle walls [9]. Furthermore, compared to other effective materials, like glass, FLPE bottles are splinter-proof and have tight-fitting caps. However, a rigorous cleaning protocol is required to pristine them and remove any Hg trace, even those given by their factory production process. In our study, we propose a new customized cleaning protocol for 500 mL FLPE bottles, which is an edited version from that suggested in the EPA method 1631E. This procedure consists of 10 steps (Fig. 1), which involves the use of an orbital shaker (e.g., KS 501 digital by IKA^Ⓡ^-Werke GmbH & Co. KG, Germany) to enhance the stirring process. The method workflow is the following:

(1) Leach in an alkaline detergent (Micro-90, concentrated cleaning solution for critical cleaning; Cole-Parmer). Rinse thoroughly with de-ionized water.
(2) Fill up the bottle to about a third with acetone and transfer on the automatic shaker. After 30 min, pour off the solvent and rinse each bottle with ultrapure water. If this solvent would be running out, it can be saved and reused.
(3) Fill up the bottle to about a third with methanol and stir for 30 min. Rinse with ultrapure water. As for the acetone in step 2, the recovered solvent can be reused if needed.
(4) Fill up the bottle to about a third with ultrapure water and shake for 30 min.
(5) Add about 150 mL of a 3.5% v/v HNO_3_ solution and shake for at least 3 hours.
(6) Rinse with ultrapure water (about a third of the bottle) for 30 min under stirring.
(7) Fill up the bottle to about a third with a 1% v/v BrCl solution and keep under stirring overnight. This step is the most important since it aims to oxidize any mercury residue adhered to the bottle walls.
(8) Pour out the above solution in a glass bottle and save it for the next bottle batch. This solution can be reused until it turns colorless. Rinse the cleaned bottles with copious amounts of ultrapure water.
(9) Allow for the drying of the bottles in a clean area (e.g., laminar flow hood or cabinet) in Class 100 cleanroom.
(10) Tightly cap each bottle, and seal it into a double brand-new polyethylene zip-type bag. The protected bottles can be stored in wooden or plastic boxes until use.

After the cleaning procedure, 10 mL of 0.8% v/v ultra-purity HCl solution shall be added to each bottle to preserve the water sample after collection.

The absence of Hg contamination in the cleaned bottles must be investigated by the analysis of bottle blanks obtained filling the empty bottle with 250 mL of ultrapure water and 2.5 mL of BrCl (1% of the ultrapure water volume). The THg content in each bottle must be measured after about 18 hours storage inside a HEPA-filtered laminar flow hood in Class 100 cleanroom. A bottle can be considered ready for use if THg concentration is lower than the detection limit of the EPA method 1631E, which is 20 pg.

Compared to the original method, the main benefit of this procedure consists of cutting back the time needed to clean each bottle. Indeed, unlike the EPA method, whose protocol for the cleaning of the sampling equipment requires more than 48 h, our study provides for a faster procedure to obtain Hg free bottles, requiring less than 24 h. Consequently, the entire analytical process, from sampling to analysis, is less time-consuming than the original method. Also, the proposed protocol avoids the use of hot concentrated acids, such as the HNO_3_ 4N at 65–75°C reported by the EPA method, which can pose health risks to laboratory operators.

### Reagent blank contamination

Reagents involved in total mercury analyses have to be of high-quality grade to prevent any contamination. In this regard, EPA method 1631E suggests the use of reagent blanks to demonstrate that the amount of Hg is lower than the detection limit (20 pg). In detail, a reagent blank should be prepared and analyzed for each solution employed in the analysis:

• the hydrochloric acid used to preserve the samples.
• the bromine monochloride used as an oxidizing agent.
• the hydroxylamine hydrochloride used to neutralize the excess of BrCl.
• the stannous chloride dihydrate, used as a reducing agent.

The use of ultra-purity grade reagents, as for HCl, therefore should be enough to ensure the lack of contamination but for the other reagents, given the sensitivity of the method and the low concentrations we want to determine, the EPA method 1631E entitles further purification procedures including the purging of certain reagent solutions, such as SnCl_2_·2H_2_O or NH_2_OH·HCl, with mercury-free nitrogen or argon. However, a different strategy must be considered for the BrCl solution since it cannot be purified once it is prepared. Thus, in case of contamination observed during the analysis of reagent blank, a new batch shall be prepared, and tested again for potential contamination. The procedure for the preparation of BrCl, as suggested in the EPA method 1631E, requires the use of reagent grade KBr and KBrO_3_. Indeed, BrCl is prepared by dissolving 27 g of KBr in 2.5 L of ultra-purity grade HCl in a fume hood. After 1 hour stirring, 38 g of KBrO_3_ are slowly added to the acid solution leading to a color change from yellow to bright orange.

In our study, two different purity trademarks of KBr (Suprapur and ACS commercialized by Merck and Sigma Aldrich, respectively) and KBrO_3_ (EMSURE and ACS commercialized by Merck and Fluka, respectively) were tested to assess their contribution to the resulting BrCl solution. The contamination level was evaluated through the comparison of the peak area obtained from the analysis of reagent blanks of BrCl solution (BrCl aqueous solution at 0.5% v/v) with three references, i.e., the peak area given by the same instrument in optimum working conditions for reagent blanks, calibration blanks and the 0.5 ng L^−1^ calibration standard solutions.

**Fig. 2:**
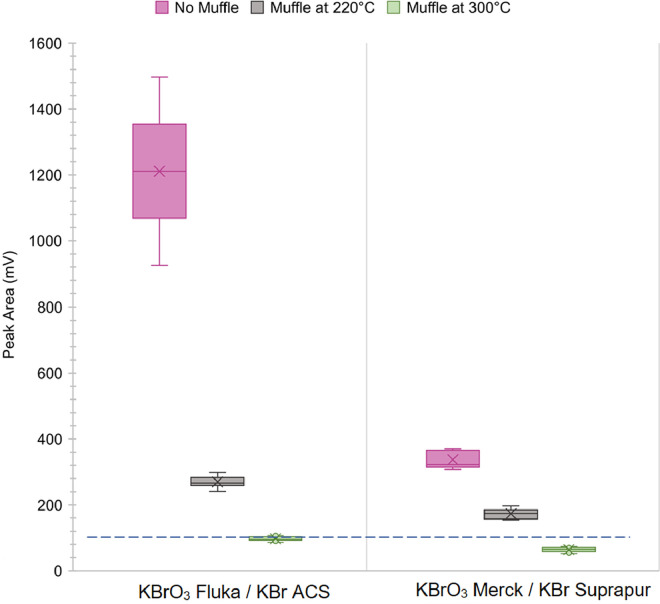
THg levels detected in a BrCl solution (0.5% v/v) before and after the thermal treatment of KBrO_3_ and KBr. The dotted line indicates the acceptable threshold for the THg signal in the blank sample.

The use of the above-mentioned purity trademarks of each salt, because of higher quality than those suggested in the EPA method, should have ensured the lack of Hg contamination. Conversely, a significant THg level was detected in the BrCl solution, as results from the high peak areas of the BrCl prepared by reaction of the ACS KBr with ACS KBrO_3_ and of the Suprapur KBr with EMSURE KBrO_3_ (Fig. 2). Consequently, we further investigated each salt by testing them individually (150 mg of salt dissolved in 50 ml ultrapure water) and we found that THg levels in the ACS KBr and KBrO_3_ were higher than Suprapur KBr and EMSURE KBrO_3_.

A potential strategy to reduce the Hg content in all the four salts involves heating them in a muffle furnace at 220°C for 48 h [10]. Unfortunately, this treatment was ineffective in the removal of contamination, and a significant amount of THg was still detected in the BrCl blank samples (Fig. 2) making it unsuitable for a successful use according to the EPA method. On the contrary, we obtained a significant reduction of the THg content after muffling each salt at 300°C for 1 week, conditions that did not affect the stability of the salts but forced the release of Hg in the gaseous phase. In this case, the analysis of the reagent blank showed the effectiveness of the thermal treatment, reducing the THg concentration in the BrCl solutions down to acceptable levels, which is a peak area value lower than 100 mV (Fig. 2). In conclusion, after that the salts have been purified, we recommend keeping them in the warm muffle to prevent the potential Hg reabsorption. Besides, in these tests special care was taken throughout the procedure to avoid contamination from external sources, e.g., from the glass crucible used as a container for each reagent. In this case, we found that effective cleaning was attained by washing the glassware with concentrated HCl (36% w/w), followed by a rinse step with a copious amount of ultrapure water.

### Control of the carryover contamination

A key role for reliable measurements is represented by the control of carryover contamination, which is challenging after repeated analyses or when high concentration samples are investigated. For a flow injection Tekran 2600, analyte carryover in the tubing system as well as over the PS may pose a treat for the next analyses. Indeed, a slight Hg contamination may result from the adhesion of Hg on the tubing walls and the frosted glass of the PS, especially when some inorganic deposits are present. An example is given by the deposition of pale-yellow residues, which occurs as a consequence of the SnCl_2_ oxidation. To tackle its formation that can potentially foster the entrapment of Hg from the samples, it is strongly recommended the use of SnCl_2_ at the lowest effective concentration.

Unfortunately, the EPA method does not provide for any cleaning procedure aimed to remove the inorganic deposits from the inside tubing walls and the PS rod. A practical recommendation for minimizing the risk of memory effect is reported in the instrument manual, which suggests running a rinse cycle at the end of the analyses by recirculating aqua regia (HNO_3_/HCl 1:3) in the tubing system from the autosampler inlet to the PS output. However, in our study, we found that the recirculation for 20 minutes of ultra-purity HCl in the sample tubing and over the PS was more effective than aqua regia for the cleaning procedure, thus allowing for rapid elimination of the deposits without the disassembling of the sampling line. Indeed, the use of HCl resulted able to dissolve the stannous deposits turning them into water-soluble species, e.g., chlorinated complexes. Similarly, in case of strong encrustation on the PS, we verified that an overnight soaking with concentrated HCl was suitable for the PS cleaning, allowing to attain an effective elimination of the deposits from the frosted glass.

### Application of the modified EPA method 1631E to the RECETOX-UNEP-Global Assessment of Laboratories Analyzing Mercury

The quality of the measurements performed by CV-AFS and the effectiveness of our cleaning procedures previously reported were assessed through the participation at the interlaboratory comparison study “Global Assessment of Laboratories Analyzing Mercury” (First Round, 2018) [11]. This study was organized in July 2018 by the UN Environment Chemicals and Health Branch and by the Research Centre for Toxic Compounds in the Environment (RECETOX), which acted as the Stockholm Convention Regional Centre in the Czech Republic. Some representative laboratories worldwide, with a geographical distribution among UN regions were invited to compare their performance analyzing THg in an aqueous standard test sample.

The involved laboratories received the test sample (i.e., standard sample) and assessed the Hg concentration using various analytical methods and instrumental detection techniques, including Cold Vapor Atomic Absorption Spectrometry (CV-AAS), CV-AFS, and Inductively Coupled Plasma Mass Spectrometry (ICP-MS).

The results of the test were gathered and finally expressed in terms of z-scores [11]. Our laboratory accomplished the blind test following our edited version of the EPA method 1631E. In detail, a new batch of BrCl was first prepared by treating the salts in agreement with the protocol previously suggested and tested through the analysis of 0.5% v/v BrCl solutions. Later, the instrument was calibrated for THg in the range 0.5 – 100 ng L^−1^ using working standards prepared by dilution of a standard stock solution. After preliminary tests, five replicates were prepared by dilution of the standard sample so that the associated signal resulted within the linear dynamic range of the method. Then, the diluted standard samples were spiked with 0.5% v/v BrCl and left overnight under a fume hood. Each replicate was analyzed, and the results were averaged.

According to our analysis, the mercury concentration in the standard sample was 20.2 ± 0.8 µg L^−1^. As illustrated in the preliminary report “Global Assessment of Laboratories Analyzing Mercury, First Round”, our concentration value, corresponding to the Lab. n. 159, resulted in a good agreement with the actual concentration value (z-score 0.80), thus suggesting the effectiveness and comparability of our analytical method. The outcomes of this interlaboratory comparison have proved to be useful to assess the performances of the worldwide involved laboratories in mercury analysis, which are essential in support of the requirements of the Minamata Convention on Mercury.

### Concluding remarks

Accurate determination of trace levels of mercury in aqueous matrices is a complex task that requires strict control of the possible contamination sources as well as particularly sensitive instruments. In this study, we proposed a customized version of the EPA method 1631E for the Hg analysis in aqueous samples using a Tekran 2600 analyzer. In our edited version, we provided procedures for the cleaning of sample containers and the minimization of the contamination risk before and after analyses. In detail, the main advancement involved a procedure for the cleaning of the sampling bottles, which allows for a significant shrink of the operating times in comparison with the protocol of the original method. Furthermore, the strong thermal pre-treatment of the commercial salts KBr and KBrO_3_ necessary for the preparation of the BrCl has proved to be an effective strategy for reducing the reagent contamination, which could potentially result in biased measurements. Even the cleaning of the instrument after the analysis cycle based on the recirculating of concentrated HCl represents an efficient approach for the control of the memory effect. All the customizations of the EPA method 1631E were successfully exploited in the interlaboratory comparison study RECETOX-UNEP-Global Assessment of Laboratories Analyzing Mercury by analyzing an aqueous standard test sample with unknown Hg concentration. Furthermore, given the basic nature of the improvements made, their use can be generalized to all aqueous samples, including those obtained by acid digestion of solid matrices.
